# Supplementary material for: Are carbon emissions trading and green financial instruments synergistic? -Comprehensive quantitative research based on content analysis
Source: PLoS One. 2024 Mar 7;19(3):e0298601. doi: 10.1371/journal.pone.0298601 (PMC10919601; doi:10.1371/journal.pone.0298601)
Supplement: S1 Data — (ZIP) [file pone.0298601.s001.zip › Data/地级市Z_F工作报告.zip!/║╙▒▒/▒ú╢¿╩╨/└┤╫╘╣1⁄2╓┌║┼í╛┬φ┐╦╩2╛▌═°í┐_2014.docx]

各位代表：

　　现在，我代表保定市人民政府向大会作工作报告，请予审议，并请市政协委员和列席会议的同志提出意见。

　　一、2013年工作回顾

　　刚刚过去的2013年，是本届政府的开局之年，也是全市经济社会发展迈出新步伐的一年。在省委、省政府和市委的坚强领导下，全市上下紧紧围绕打造环京津地区新的发展增长极，以解放思想、改革开放、创新驱动、科学发展为统揽，扎实推进稳增长、调结构、攻重点、抓改革、惠民生、优环境各项工作，较好地完成了市十四届人大一次会议确定的目标任务。

　　（一）积极应对下行压力，经济保持平稳增长。全市生产总值完成2650.6亿元（不含定州，下同）、增长9%，高于全省0.8个百分点。全部财政收入324.8亿元、增长13.1%，公共财政预算收入167亿元、增长13.9%。农业连续11年丰收，粮食总产525万吨；实现工业增加值1004.7亿元、增长11.4%，新增规模以上工业企业218家；固定资产投资增长9.6%；社会消费品零售总额增长13.8%，增速全省第一；野三坡、白石山、狼牙山等景区灾后涅槃重生，全市旅游综合收入318.8亿元、增长28.8%；农民人均纯收入8675元、增长13.2%，城镇居民人均可支配收入21181元、增长11.2%。主要指标在全省位次前移，实现稳中有进、稳中向好。

　　（二）大力调整产业结构，转型升级步伐加快。汽车产业保持快速发展，全市生产整车82.5万辆、增长18.8%，长城汽车销售收入534亿元、增长27.1%，利税126亿元、增长59.5%，销量居自主品牌车企之首。新能源产业企稳向好，英利光伏组件产量增长30.6%，居世界首位。纺织服装、食品、建材产业增加值分别增长12.6%、13.4%和17.4%。园区和项目建设快速推进。徐水大王店汽车产业园初具规模，高碑店奥润顺达节能门窗基地被认定为国家建筑节能技术国际创新园。与中科院北京分院、清华大学、北京中医药大学等达成战略合作；校地合作有新进展，全省首个市级校企联盟成立；高新技术产业快速发展，创新驱动力明显增强。

　　（三）强势推进招商引资，开放氛围日渐浓厚。举办了北京、天津招商周，是近年来影响最大、收获最多、效果最好的一次招商推介。廊坊经洽会、厦门推介会、广州交易会取得丰硕成果。举办侨商、台商、外商、民企进保等推介活动10余次，与商务部投资促进局和贸发局建立战略合作。涞源中美科技创新园、正威曲阳雕塑文化产业新城、新发地高碑店农副产品加工园区、航天科工涞水科技研发基地等一批项目落户我市。实际利用外资6.2亿美元、增长36.7%，增速全省第一。

　　（四）重拳出击创优环境，四项行动成效明显。开展城市管理“6S”行动，向违法建设、垃圾死角、马路市场、露天烧烤等城市顽疾全面宣战，声势之大、范围之广、措施之硬前所未有，市容市貌明显改观。发起全民创业总动员，出台30条突破性政策，多措并举打造创业之城，市场主体总量达到26.5万户、增长8.4%，民营经济增加值1805.6亿元、增长9.8%。强力推进效能建设，市本级审批事项大幅削减，行政服务中心压减办理环节，承诺时限整体提速，群众满意度有较大提高。开展“秋收100”专项行动，利剑出击，打掉各类犯罪团伙837个，破获刑事案件17092起，其中破坏发展环境案件6347起，打出了气势，打出了声威，打出了浩然正气和崭新形象，我市经验在全省推广。

　　（五）聚焦聚力攻坚克难，重点工作取得突破。以壮士断腕的决心打响大气污染防治攻坚战，全民动员洗城净天，淘汰取缔燃煤锅炉776台，关停土小企业4757家，淘汰“黄标车”4.5万辆，是全省措施最硬、力度最大的市之一。以人为本、过细工作，集中力量推进重大项目征迁，京港澳高速拓宽、保津城际铁路、南水北调配套工程等顺利实施，特别是仅用100天就完成大王店工业园区1.4万亩土地、5个村庄的整体征迁，农民高兴，企业满意，省委省政府充分肯定。扎实开展农村面貌改造提升行动，全力推进灾后重建和扶贫攻坚，阜平、曲阳扶贫开发模式全省推广。着力破解土地和资金瓶颈制约，保定银行顺利完成第二次增资扩股，新增银行、保险、证券等金融机构14家，挂牌上市企业7家，为历年最多。三大片区改造实现突破，首批回迁安置顺利实施。吸引民营投资完成直隶大剧院改造，为保定老调、河北梆子等院团解决了办公排练用房。宝硕、依棉、外贸印刷厂、金昌大厦等一批历史遗留难题得到妥善解决。

　　（六）倾心倾力保障民生，社会事业全面进步。52项惠民实事基本完成。城乡居民社会养老保险参保率达到96.8%。城镇居民医疗保险补助、新农合补助、城乡低保标准全面提高。社区服务热线和老年人紧急救助服务系统成为全国示范典型。万博广场、华创广场投入使用，东风路东延直通高铁车站，完成市区71条小街巷改造，申办成功全国“公交都市”试点；农业生态园展现新貌，国家园林城市正式授牌。县城广场、道路、集中供热、农村公路等建设加快。城市保障房竣工2.5万户，农村危房改造完成3万户。新解决71万农村人口饮水安全问题。义务教育标准化学校建设扎实推进，校安工程改造成效显著。基层医疗卫生机构全部实施基本药物制度。开展“文化润古城”活动，举办大中型文艺演出60余场次，文化生活日渐丰富。讲好保定故事，弘扬正能量，推出“油条哥”等一批善美典型。成功举办国际空竹艺术节、自由飞行周、世界女子兵乓球攻削大战、民俗文化节等文体活动，提高了保定美誉度。低生育水平保持稳定。安全生产和食品药品安全工作得到加强。统计、审计、档案、史志、人防、气象、外事侨务、民族宗教、防震减灾、国防动员、妇女儿童、残疾人等工作都取得新成绩。

　　（七）加强政府自身建设，工作作风明显转变。修订完善了政府工作规则，市本级和16个县（市）政务服务标准化建设通过ISO9001质量管理体系认证，市县乡三级政务服务网络框架基本形成。严格落实中央“八项规定”和各项“禁令”，对办公用房、吃空饷、公务用车等开展专项治理，廉政建设和反腐工作得到加强。坚持依法行政，自觉接受市人大、政协和新闻媒体监督，办理人大代表建议、政协提案524件，办结率、满意率100%。市长、副市长全部开通电子信箱，全年受理群众来信来电、短信息和网络留言90863件（条），交办率100%。在全省设区市中首个开通政府官方微博，累计访问量1631万次，成为沟通政情民意的新平台。

　　各位代表！过去的一年，面对近年来少有的复杂严峻经济形势，面对愈加突出的大气环境污染压力，面对艰巨繁重的保发展、保稳定、保民生任务，我们始终坚持立党为公、执政为民，殚精竭虑、奋力前行，经受住了考验，成绩来之不易。这是省委、省政府和市委科学决策、正确领导的结果，是市人大、市政协有效监督、大力支持的结果，是各级各部门勇于担当、聚力攻坚的结果，是全市上下团结拼搏、不懈奋斗的结果。在此，我代表保定市人民政府，向全市人民，向人大代表和政协委员，向中央、省驻保单位，向驻保部队和武警官兵，向社会各界人士和离退休老同志，向所有关心支持保定发展的海内外朋友们，表示衷心的感谢和崇高的敬意！

　　我们也清醒地看到，我市经济社会发展中还存在不少矛盾和问题，政府工作与人民群众的期待还有差距。尽管去年经济运行结果比预料的要好，但经济下行的压力依然很大，部分企业经营困难，工业经济增速持续放缓，项目和投资不足，县域经济不强、发展不快的问题还很突出；高端产业比重小，大企业、大集团少，中小企业层次低，节能减排压力大，产业转型升级和大气污染治理任务艰巨；经济外向度不高，园区建设相对滞后，吸引力不强、承载力不足。中心城市、县城和小城镇规模偏小、建设水平偏低，城镇化率低于全省、全国水平；古城改造和西大街整治还没有达到预期效果，关汉卿大剧院和博物馆建设因资金问题未能竣工，殡仪馆搬迁因选址问题没有明显进展，市容市貌仍有死角，城市管理还有许多不尽如人意之处。就业难、看病贵、社保水平低，部分群众生活还比较困难；教育、卫生资源不均衡等问题依然存在。政府部门和公务人员服务至上、亲商重商意识还有待强化，思想不解放、体制不灵活、办事效率不高的问题依然突出；特别是庸懒散、奢私贪、冷横硬等问题还需治理，有令不行、有禁不止，上有政策、下有对策等现象还时有发生等等。这些矛盾和问题，既是影响发展的症结，也是必须击破的壁垒。我们一定增强忧患意识、责任意识，强化问题导向、底线思维，以更大的决心、更大的力度、更有效的措施认真加以解决，决不辜负全市人民的期望和重托。二、2014年工作总体要求

　　今年是落实党的十八届三中全会精神、全面深化改革的第一年，是发力崛起、跨越发展的重要一年，是关系长远、决定未来的关键一年。全面深化改革的系列政策陆续出台，必将释放巨大的改革红利；京津冀协同发展上升为国家战略，受到高层关注，京津保三角格局得到认同；我市的区位、资源、产业优势日益凸显，正在成为承接首都功能疏解和产业转移的首选地，为我们融入京津冀城市群、打造环京津新的增长极提供了前所未有的重大机遇。特别是全市上下政通人和、人心思进，其势已成、其力已聚，只要我们坚定信心、乘势而上，必将激发巨大的正能量，开创保定发展的新辉煌。

　　今年政府工作总的指导思想是：全面贯彻落实中央、省、市一系列会议精神，牢牢把握京津冀协同发展大势，紧紧围绕发展稳定民生三大任务，坚持“点线面”整体推进、“六个主战场”全面发力，改革创新、高端发展、真抓实干，着力在对接京津、项目落地、产业升级、城乡统筹、扶贫攻坚、优化环境上打开新的突破口，努力实现打造环京津增长极的新突破，建设人民富裕的保定、生态文明的保定、风清气正的保定。

　　今年经济社会发展的主要预期目标是：生产总值增长9%左右，规模以上工业增加值增长12%以上，固定资产投资增长18%，社会消费品零售总额增长13%以上，公共财政预算收入增长11%。单位生产总值能耗和化学需氧量、二氧化硫、氨氮、氮氧化物排放量完成省定目标，细颗粒物（PM2.5）浓度下降4%。城镇居民人均可支配收入和农民人均纯收入分别增长10%，居民消费价格指数涨幅控制在3.8%左右，城镇登记失业率控制在4.5%以内，人口自然增长率控制在7.8％。以内。

　　做好今年工作、实现预期目标，必须把改革创新的动力贯穿始终、把高端发展的主线贯彻始终、把真抓实干的作风贯穿始终。坚定不移抓发展。始终把发展放在第一位，努力保持一定的发展速度；着力推动产业升级，全力推动高端发展，坚决走绿色崛起之路。坚定不移抓改革。打破体制机制束缚，以改革促创新、促转型，使发展潜力充分释放、发展动力进一步增强。坚定不移抓开放。强力实施“开放兴保”战略，放开手脚、敞开胸怀，打造开放平台，持续掀起招商引资热潮，做实项目根基，提高区域竞争力。坚定不移抓环境。全面改善政务环境、生态环境、城市环境、社会环境，打造创业之城、创新之城、低碳之城、宜居之城，以良好环境保障跨越发展。坚定不移抓民生。牢记民生为先、人民为重，持之以恒为群众办好事办实事，努力让群众得到更多实惠、过上更美好的生活。

　　三、聚力打造环京津增长极，做强跨越发展的支撑

　　紧紧围绕服务京津、承接京津、融入京津，突出“开放、高端、创新、统筹、效率”，推进重点区域、重点产业、重点项目率先突破，提升保定在京津保三角中的地位和份量。

　　强力推进与京津对接融合。把承接北京功能疏解和产业转移作为保定跨越发展的引擎和引爆点，以“一核两组团”为重点区域，打造首都功能疏解的支撑点、京津产业转移的承接地、外资进入京津乃至北方市场的桥头堡。突出抓好“一城、一基地、一区、一体系”：一是河北白洋淀科技城。依托驻保高校资源、高铁交通优势和白洋淀生态品牌，在保定市东部区域，规划建设河北省科技创新平台，加强与北京高等院校、中关村和国内外科技型企业战略合作，打造产学研一体化发展示范基地、新兴产业基地、改革开放示范区和创新发展的新引擎。二是京南现代产业基地。加快以保北低碳新城为核心的汽车、新能源、智能电网等高端装备制造基地建设，加快高碑店节能环保产业基地、涿州和涞水、涞源战略新兴产业基地以及涿州义和庄空港产业基地建设，推进白沟和道国际箱包交易中心、高碑店和定兴农副产品专业市场以及市区、安国、容城等重点现代物流基地建设，做强对接京津的产业支撑。三是首都服务功能承接区。加快打造以保定、涿州、高碑店三个高铁新城为重点的现代服务业集中区、以主城区和涿州等为重点的医疗养老服务集中区、以白洋淀和涞涞易为重点的旅游休闲度假基地，建设高等教育、医疗卫生、养老服务功能承接区。四是现代快速交通体系。抓好津保城际、廊涿城际、固安至白沟新城城际铁路和京港澳高速拓宽、京昆高速北延、荣乌高速贯通等重点交通工程，完善配套骨干路网，打造京南交通陆路枢纽，加快与京津无障碍、无缝隙对接和同城化发展。

　　承接北京功能和产业转移，京津冀协同发展，这是保定面临的重大机遇，也是落在我们肩上的历史责任。全市上下要树立强烈的“抢先”意识，各县（市、区）和各部门要积极主动作为，做好充分准备。要超前谋划、抢占先机、抢抓机遇、争先恐后，切不可等待观望、反应迟钝、坐失良机，不能留下历史的遗憾，更不能愧对我们的子孙后代。

　　强力抓好园区和项目建设。把园区和项目建设作为跨越发展的根本着力点，努力实现新突破。推进园区上档升级。加快推进大王店工业园、涞源中美科技创新园等34个园区建设，大力提升园区承载能力。创新管理体制，鼓励社会力量参与园区（开发区）建设管理经营，打造更多产业发展的高端平台。加强重点项目建设。安排市以上重点项目200项左右，总投资3000亿元以上，年投资600亿元以上。加快长城汽车新技术中心、乐凯新材料、向阳航空产业园、清苑中汽零产业基地和解放军总医院涿州保障基地等一批在建项目进度，确保涞水悦康制药、四方三伊智能电网、中兴汽车扩能、涿州健康立体城、高阳现代农机装备等一批项目开工建设。各县（市、区）、开发区精心谋划一批超50亿元、百亿元大项目。积极争跑项目，做实项目服务，加快项目转化，让开工项目为固定资产投资添秤，竣工项目为经济发展早做贡献。

　　强力打造保定产业升级版。按照12大产业发展路线图，实施产业升级、技改升级、龙头带动和创新驱动工程，加快构筑多元支撑的现代产业体系。推进工业加快上档升级。大力发展汽车、新能源、电子信息、生物医药、新材料、节能环保等新兴产业，改造提升纺织服装、食品、建材等传统产业，实施龙头带动计划和产业链升级工程，抓好省千项技改项目落实。对列入省“三个一百”的企业加大扶持力度，力促长城、英利、巨力、奥润顺达、晨阳、九孚等企业做大做强。各县（市、区）、开发区分别筛选一批行业龙头、一批高成长性企业予以重点扶持。帮扶中小微企业，支持科技型中小企业，推进工业企业加快发展，年内新增规模以上企业200家以上，建设实力保定。提升服务业规模和水平。大力发展旅游业，白洋淀、野三坡创建5A精品景区，白石山等五处景区升格5A，加快易水湖等景区景点建设，打造名山、名水、名文化。大力发展现代物流业，实施电子商务三年发展推进计划，推进“宽带中国”和“智慧城市”建设，实施城市百兆光纤工程、县级宽带乡村工程和物联网应用示范工程。大力发展金融服务业，鼓励民间资本进入金融服务领域，支持金融机构在县城增设网点、村镇银行，发展壮大担保公司、产业基金。继续引进民生银行等股份制银行，做大做强保定银行并力争在石家庄设立分支机构，优化金融生态环境。推动宏州石业、古城香业、奥润顺达等企业上市融资，各县（市、区）、开发区每年至少新增一家企业挂牌上市。建立覆盖全面的信用数据库和共享平台，推行失信“黑名单”制度，建设诚信保定。支持文化产业加快发展。巩固提升传统文化产业，培育发展文化创意、动漫游戏、影视制作等新型业态，加快新市区数字印刷产业园、清苑正迪欢乐世界文化创意园、涿州中华文化小镇、顺平尧文化博物馆、定兴实甫文化创新产业示范园、易县易水砚文化产业园、涞水祖冲之文化园等建设，发展安新芦苇、顺平桃木等特色手工艺品，繁荣文化市场，壮大文化企业，打造文化精品，叫响文化强市，建设文化保定。

　　四、扎实做好“三农”工作，强农富民做美农村

　　保定要强，农业必须强；保定要富，农民必须富；保定要美，农村必须美。促进农业现代化与工业化、信息化、城镇化同步，夯实保定跨越发展的基础。

　　努力让农业强起来。发挥资源、区位优势，瞄准京津市场，着力调整农业结构、提升产品品质、增强竞争实力，促进现代农业加快发展。稳粮食。突出抓好12个粮食生产基地县、400万亩核心区建设，总产稳定在500万吨以上。建园区。围绕进一步做大做强畜牧、蔬菜、林果、中药材等产业，建设一批现代农业示范园区，每个县（市）年内至少打造一个5000亩以上园区，环首都县（市）建设一批京津市场供应基地。提品质。推广农业节水灌溉、无公害种植、规模化养殖、动植物防疫等技术，控肥、控药、控添加剂，规范生产过程，健全农产品质量安全追溯体系。强龙头。以工业理念发展农业，培育壮大龙头企业，加强农超对接和直营店建设，延伸农业产业链条，全市农业产业化率达到65%。重点扶持龙头企业，支持望都蒙牛、顺平汇源、定兴喜之郎和西麦、高碑店康师傅和娃哈哈、雄县雏鹰等一批领军企业做大做强，加快农副产品批发市场和物流园区建设，培育一批知名品牌，提高京津市场占有率。

　　努力让农民富起来。严格落实各项农业补贴政策，完善粮食主产区利益补偿机制，实施粮食最低收购价制度，稳定农民务农收入。支持农民土地流转，引导工商资本投资发展农业，支持种养大户组建股份合作组织，发展壮大供销社，提高农民组织化程度，增加农民财产性收入。鼓励发展农村家庭手工业、生产资料配送等多种经营，提高农民社会化收入。实施农村劳动力转移培训阳光工程，严格农民工工资支付监管，保障农民务工收入。

　　努力让农村美起来。着力改善农村生产生活条件。加强农田水利设施建设，抓好农村基础设施建设，积极推广沼气等清洁能源，实施农村电网改造升级工程，继续解决好农民群众饮水安全问题。抓好农村人居环境整治，重点推进道路硬化、村庄绿化、垃圾处理、危房改造、厕所改造，坚决改变农村脏乱差局面，年内完成361个重点村改造提升任务，加快环首都县（市）整体推进步伐，努力建设美丽乡村、幸福家园。

　　五、加快新型城镇化进程，促进城乡一体化发展

　　把城镇化作为跨越发展的重要载体和实现现代化的战略任务，着眼融入京津冀城市群，推进城镇组团发展、集群发展，努力走出一条以人为本、优化布局、生态文明、传承文化的新型城镇化道路。

　　加快中心城市拓展提升。加快推进“一城三星一淀”大保定建设，增强区域发展的承载力、带动力。拓展空间。以北部低碳新城和东部高铁新城为支点，加快中心城市向四周扩张。按照同城化发展思路，加快城市主次干道和城星通道建设，启动实施复兴路西延、东风路西延、乐凯大街南延、北三环东延西延、白洋淀大道等骨干道路建设，进一步优化空间布局，促进徐水、清苑、满城、安新与主城区协同发展。完善功能。加快棚户区和城中村改造步伐，加快推进三大片区拆迁建设和回迁安置。进一步完善供热供气、便民市场、体育休闲、垃圾处理、地下管网、防灾减灾等基础设施，建设一批老百姓身边的市政工程。提升形象。加快北市区东湖片区、新市区西湖片区、南市区长城科技城开发和古城新天地商旅文综合体验区建设，完成万博广场、燕赵新天地、火车站综合改造等重点工程建设，浓郁城市现代气息；完成中国衙署文化博物馆、关汉卿大剧院和博物馆建设，加快直隶总督署、淮军公所、清河道署、直隶审判厅修缮及周边整体拆迁改造，彰显城市古韵文脉；抓好亮化绿化、城市雕塑、水系景观等城市美化工程，启动动物园搬迁，完成竞秀公园等公园游园改造和漕河景观建设，打造城市靓丽风景。

　　推进城镇建设扩容提质。以创建省级园林县（市）城为引领，按照坚守特色、完善功能和“七个一”要求，实施一批基础设施、园林绿化、景观风貌工程，力争使每个县城做到“三有四达标”。涞源、曲阳、唐县、望都等17个有河道的县城，两年内做好水文章。积极推进白沟、涿州松林店、蠡县留史、高阳庞口、定兴固城、涞水石亭等国家和省级重点镇建设，重点培育30-50个工业型、旅游型、商贸型、交通型明星城镇，每个县（市）集中打造1-2个特色鲜明、充满活力、生态宜居的示范镇。加强历史文化名村、古村落保护与开发，构建城市、城镇、乡村一体化发展格局。

　　积极推动产城互动发展。坚持以产兴城、以城带产，按照布局集中、产业集聚、用地集约的要求，完善城镇功能分区，引导产业向园区集中，园区向城镇集中。坚持特色兴县，分类指导、因地制宜，大力发展县域经济，努力实现“二次创业”。每个县重点培育壮大1-2个特色主导产业，重点支持安国中药、曲阳雕刻、博野输送带、蠡县皮毛、容城服装、白沟箱包、高阳纺织等县域特色产业转型升级、做大做强，支持徐水、安国打造千亿元产值和营业收入县（市）。进一步活跃城市经济，汇聚人流、物流、信息流，使农村转移人口进得来、留得住、过得好。

　　全面提升城镇管理水平。树立尊重自然、顺应自然、天人合一的规划理念，坚持经济社会发展、城乡、土地规划“三规合一”，进一步完善城镇规划体系，刚性执行规划，坚决杜绝新的违法建设。坚持集约节约利用土地，科学合理布局，努力避免交通拥堵、环境恶化等城市病。深化城市管理“6S”行动，由突击性治理向长效性管理转变、由靠部门执法监管向全民自觉行动转变、由政府包办向市场化运作转变；由市区向县城和乡村延伸，由城市主干街道向小街小巷和社区延伸，真正做到无缝隙、全覆盖、零死角，让城市净起来、亮起来、美起来。六、铁腕治理环境污染，全方位加强生态建设

　　以更大力度抓好污染治理、环境保护和生态建设，让人们在更好的环境中生活，增强保定在京津冀城市群中的生态魅力。

　　强力推进大气污染防治。以“压煤、抑尘、控车、迁企、减排、增绿”为主线，坚决打好大气污染防治攻坚战、持久战。大力压减煤炭消耗，加快推进集中供热建设，取缔、淘汰和改造燃煤锅炉，加强煤炭质量监管，严禁不达标劣质燃煤进入市区。推广农村秸秆压块技术，减少农村燃煤用量。全面禁止焚烧秸秆、树叶、垃圾。坚决抑制扬尘污染，所有在建工地实行绿色施工，所有露天矿山、采砂场实行绿色生产，所有沙石、渣土运输车辆做到无洒漏。严控机动车污染治理，加强成品油质量监管，加强机动车污染检测，淘汰所有“黄标车”。狠抓节能减排，推进重点行业污染减排，搬迁市区重污染企业，加快工业、交通、建筑等重点领域节能改造。始终保持高压态势，坚决取缔各类土小企业，严防死灰复燃。加大环境执法力度，对一切环境违法行为“零容忍”、严厉惩处。加强科技治污，抓好宣传引导，加强重污染天气应急管理。通过多措施治，力争空气质量三年好转、五年明显改善，还人民一片洁净蓝天。

　　着力抓好生态环境修复。启动山水林湖田生态修复计划，加强白洋淀及上游污染治理，谋划实施淀区居民搬迁，打造环首都生态示范区和功能区。加大西大洋、王快等水库水源地保护力度。加快南水北调、引黄补淀等配套工程建设，严控地下水超采，提高水资源支撑和可持续利用能力。加强耕地保护，减少面源污染，加快土壤污染治理和修复。创建国家森林城市，实施三年增绿计划，全市森林覆盖率每年提高1.3个百分点，三年内达到26.5%，努力让人们望得见青山、看得见绿水，生活在天蓝水清、绿满城乡的美丽家园。

　　深入推进低碳城市建设。继续抓好“国家低碳城市”试点，争创全国新能源示范城市。深化“太阳能之城”建设，大力实施分布式太阳能电站建设，打造沿太行山光伏规模化应用示范带。推进“气化保定”工程，启动新能源汽车试点项目。科学开发利用地热资源，在雄县、博野等县探索建设“无烟城”。鼓励发展绿色交通，倡导绿色出行，积极推进全国“公交都市”试点建设。深入开展低碳社区（小区）、低碳园区、低碳企业等创建活动，倡导低碳生产生活方式。

　　七、深入推进改革开放，增强发展的动力和活力

　　把改革开放作为保定跨越发展的动力之源、活力之本，坚定不移推进，努力取得突破。

　　全面深化各项改革。重点推进四项改革攻坚：深化行政体制改革。围绕转变职能，以简撤为主、减放结合，制定并公开行政审批事项目录，目录之外的一律停止审批。推动市向县（市、区）、县向乡（镇）下放权力，该放的一放到底，该减的一项不留。深化投资体制改革。打破行业垄断，鼓励社会资本进入城镇基础设施、基础产业、公共事业等领域，吸引民营企业参与投资建设、经营和管理。选择15个部门、84项服务作为试点，向社会购买公共服务。深化国有企业改革。探索组建不同类型的国有资本运营管理平台，积极发展混合所有制经济，鼓励支持国有资本、集体资本、非公有资本等交叉持股、融合发展。加快槐茂酱菜厂等企业改制步伐。深化农村综合改革。严守耕地红线，赋予农民对承包地的占有、使用、收益、流转及承担经营权抵押、担保权能。稳步推进农村产权改革试点，建立、规范市县级农村产权流转交易市场。鼓励发展家庭农场、农民合作社、农业公司等新型农业经营主体。

　　实施开放兴保战略。广开合作大门、广交天下朋友、广聚八方资源、广纳四海贤才。千方百计引进来，瞄准世界500强、中国500强企业，开展产业链招商、园区招商、精准招商、以商招商、常态化驻点招商。瞄准京津，继续开展央企进保、外企进保、大型民企进保、总部进保、人才进保、新技术进保六大攻坚行动，不断拓展深度和广度。面向长三角、珠三角等地区和香港、台湾、新加坡、美国、德国等有合作基础的国（境）外地区，举办专题招商推介会和特色产业展会，精心筹备参加廊坊、厦门等投洽会。积极主动走出去，积极拓宽外贸市场，抓好安国中药等国家级出口基地和一批服务外包示范园区、产业基地、农产品出口示范县建设，支持汽车及零部件、光伏等优势产品出口，提升外向型经济水平。

　　打造创新创业之城。把改革开放的成果落实到创新创业上、落实到加快发展上。深化创新驱动战略，加大科技创新投入，集中资金、土地等要素向科技型企业倾斜。鼓励支持骨干企业开展技术研发和产品创新，扶持科技型企业做大做强。加强产学研合作，推动优势产业、骨干企业与大专院校、科研单位共建创新平台，促进科技成果转化。围绕“品牌兴市、人才兴市”，推出一批竞争力强、叫得响的名牌产品和商标，培养和引进一批科技领军企业、领军人才。深化全民创业行动，实施市场主体增量计划，进一步降低准入门槛，实行投资主体“零限制”、注册服务“零收费”，由“先证后照”改为“先照后证”。抓好高新区大学科技园、北市区大学生创业基地建设，新建一批中小企业孵化基地，让百姓创业便利更多、成本更小。弘扬创新创业文化，尊重劳动、尊重创造、尊重企业家，支持企业二次创业，鼓励干部创事业、能人办企业、百姓创家业，对创新企业、纳税大户、创业明星、杰出人才给予重奖，让保定成为创新创业者的乐园。

　　八、加快发展社会事业，倾力保障和改善民生

　　把民之所望作为施政所向，优先保障民生投入，优先安排民生项目，优先解决民生问题，让发展成果更多更公平惠及全市人民。

　　健全社会保障。实施就业优先战略，全力做好高校毕业生、农村转移劳动力、城镇困难人员、退役军人、下岗再就业职工等重点群体就业工作。扩大社会保险覆盖面，全面实施基本医疗保险市级统筹，企业退休人员基本养老金增长10%，城乡居民医疗补助标准提高到320元，城乡低保应保尽保并逐步提高标准。建设保障房3.4万套，提高廉租房、公租房比例。发展养老服务业，健全社会救助和留守儿童、妇女、老人关爱体系。

　　发展社会事业。以教育、卫生、养老等为重点，以改革创新精神推动各项社会事业加快发展。统筹各类教育协调发展，鼓励支持社会力量办学，推进义务教育公办学校标准化建设，加快保定职业技术学院迁建，支持驻保高校与地方融合发展。开展城市医院对口帮扶乡镇卫生院工作，实施县级中医院和妇幼保健院改造、乡镇卫生院升级达标工程，建立困难群体大病医疗救助机制，加快市第一中心医院门诊综合楼和区域医疗中心建设，加快市儿童医院、市妇幼保健院和养老机构建设。加快殡仪馆搬迁进程。实施文化惠民工程，推进县级文化馆、图书馆、数字影院和乡（村）文化站（室）建设，扩大“文化润古城”覆盖面，支持直隶大剧院和老马号书场做好做强，丰富群众文化生活。完善城乡公共体育设施，谋划建设体育健身主题公园。落实“单独两孩”政策，提高出生人口质量。加强民族宗教事务管理，促进民族团结进步。深入开展以“讲诚信、懂规则、守法纪”为主要内容的精神文明创建活动。加强国防教育和国防后备力量建设，推进军民融合深度发展。搞好第三次经济普查。做好气象服务、防震减灾、外事侨务、史志档案等工作，促进社会事业全面进步。

　　推进扶贫攻坚。紧盯目标、聚焦难题，深入推进“十大扶贫攻坚战”，力争阜平区域发展与扶贫攻坚试点取得阶段性成效，涞源、涞水、易县、望都、博野率先完成减贫目标，唐县、顺平、曲阳提前完成“十二五”脱贫任务。选择100个贫困村建档立卡，实施精准扶贫。加大教育、卫生对口帮扶力度，提高贫困地区公共服务水平。按照市场化运作思路，推进“三个十”工程，重点发展10大扶贫产业、打造10个太行山综合开发产业片区、建设10个现代农业园区，确保年内300个村、20万人实现脱贫。

　　建设平安保定。毫不放松抓好安全生产，突出道路、消防、矿山、危爆物品、特种设备等领域安全监管，坚决遏制重特大事故发生。加快完善市县乡食品药品安全监管体系，充实基层监管力量，实施食品药品安全县创建工作，用最严谨的标准、最严格的监管、最严厉的处罚、最严肃的问责，确保人民群众“舌尖”上的安全。推进“天网”工程建设，实现城乡高清摄像头全覆盖。坚持“一年四打、连打三年”，持续打击村霸、路霸、渣痞、欺行霸市等黑恶势力犯罪，持续打击封门堵路、敲诈勒索等破坏发展环境犯罪，持续打击“两抢一盗”等侵财犯罪，进一步增强群众的安全感。加强社会管理创新，高度重视信访工作，对群众深恶痛绝的事零容忍，对群众急需急盼的事零懈怠。维护社会和谐稳定，促进社会公平正义，保障人民安居乐业。

　　办好惠民实事。围绕解决群众最关注、最忧心、最迫切的身边问题，办好一批实事。主要包括：新建34所山区寄宿制学校，三年建成70所；资助贫困学生10万人，1020所学校接入宽带网络；为6万对农村夫妇提供免费孕前体检；对36个老旧小区供热设施维修改造；新改迁建3所便民市场，改造24条小街巷；在市区推行公共自行车服务；新增150个早餐安全示范点，加强“小饭桌”安全监管，等等。对于这些实事，我们将逐项明晰责任，统筹财力，坚决办好。

　　九、认真改进工作作风，建设为民务实清廉政府

　　对人民负责、让人民满意，建设为民、务实、清廉政府，是我们的不懈追求。做好今年的政府工作，我们要高扬“实干兴市、清正为民”主旋律，大兴实干之风，大兴创新之风，大兴清廉之风，以改革创新精神加强政府自身建设，提振精气神、全面转作风，汇集正能量、聚力抓落实。

　　深入群众，改进作风。扎实开展教育实践活动，聚焦“四风”问题，敞开大门，广泛听取意见。坚持从市政府领导班子做起，带头深入基层调查研究，带头坚持为民服务在一线，带头廉洁自律，带头改进会风文风。抓好问题整改，坚持从小事抓起，从具体问题做起，从群众最关心的事情干起，着力解决发生在群众身边、损害群众利益的问题，让群众看到实实在在的成效，努力建设人民满意的服务型政府。

　　优化环境，提高效能。进一步转变政府职能，着力为各类市场主体创造公平公正、竞争有序的市场环境。支持各类行业协会、异地商会开展工作，建立政府与企业沟通联络、主动服务的有效机制。深化效能提升行动，健全市县乡行政服务中心、村服务代办点等实体平台，谋划启动市民服务中心，真正建成“办事处”、不搞“传达室”。严格落实企业宁静日等制度，推行“限时限次办结制”、“承诺审批制”、“两不见面代办制”、企业和项目环境综合治理机制，努力建设环京津地区行政成本最低、商务成本最低、生活品质最高、服务品质最高的城市。

　　科学行政，依法行政。畅通民情民意渠道，整合22条政务热线，开通办好961890为民服务热线，政府系统全面开通政务微博，并实现衔接互动。成立政府决策咨询委员会，健全重大行政决策专家咨询、风险评估、合法性审查和民生事项听证会等制度，重大规划尊重专家意见，重要决策会议邀请人大、政协参加。推行政府工作部门权力清单制度，建设政务服务电子监察平台，让权力在阳光下规范运行。自觉接受人大及其常委会的法律监督和工作监督，自觉接受人民政协的民主监督，重视发挥新闻媒体和社会公众的监督作用，切实把政府工作纳入法制轨道，努力建设法治政府。

　　干净干事，清正廉洁。严格执行“八项规定”和廉洁自律各项规定，正确行使党和人民赋予的权力，严守官商两道、清水之交，做到敬畏社会舆论、敬畏人民群众、敬畏党纪国法。厉行勤俭节约，反对铺张浪费；严格规范会议、文件、接待、用房、公车、出差、出国等方面的管理；严格财务预算、核准、审计，从餐桌不上烟和酒、开会不发本和笔等小事做起，严控“三公”经费支出，自觉过紧日子，把纳税人的钱更多地用在事业发展和民生改善上。把权力关进制度的笼子，以公开促公正，以透明保廉洁，增强主动公开、主动接受监督的意识，让暗箱操作没有空间，让腐败行为无法藏身。做到干部清正、政府清廉、政治清明。

　　各位代表！保定正处于一个千载难逢的重要机遇期。重返第一方阵，重塑大市尊严，让人民过上幸福舒适的好日子，既是全市人民的期盼，也是各级政府的责任。我们一定紧密团结在以习近平同志为总书记的党中央周围，在省委、省政府和市委的正确领导下，秉承“鞠躬尽瘁为百姓，殚精竭虑谋发展”的为政理念，弘扬驰而不息的龙马精神，为人民着想，对历史负责，接地气，重实效，务实务实再务实，实干实干再实干，奋斗不止，昂扬向上，勇于开拓，敢于担当，和1100万英雄智慧的保定人民携起手来，努力建设京畿强市、善美保定，加快打造环京津地区新的发展增长极，共同创造保定历史的新辉煌！
